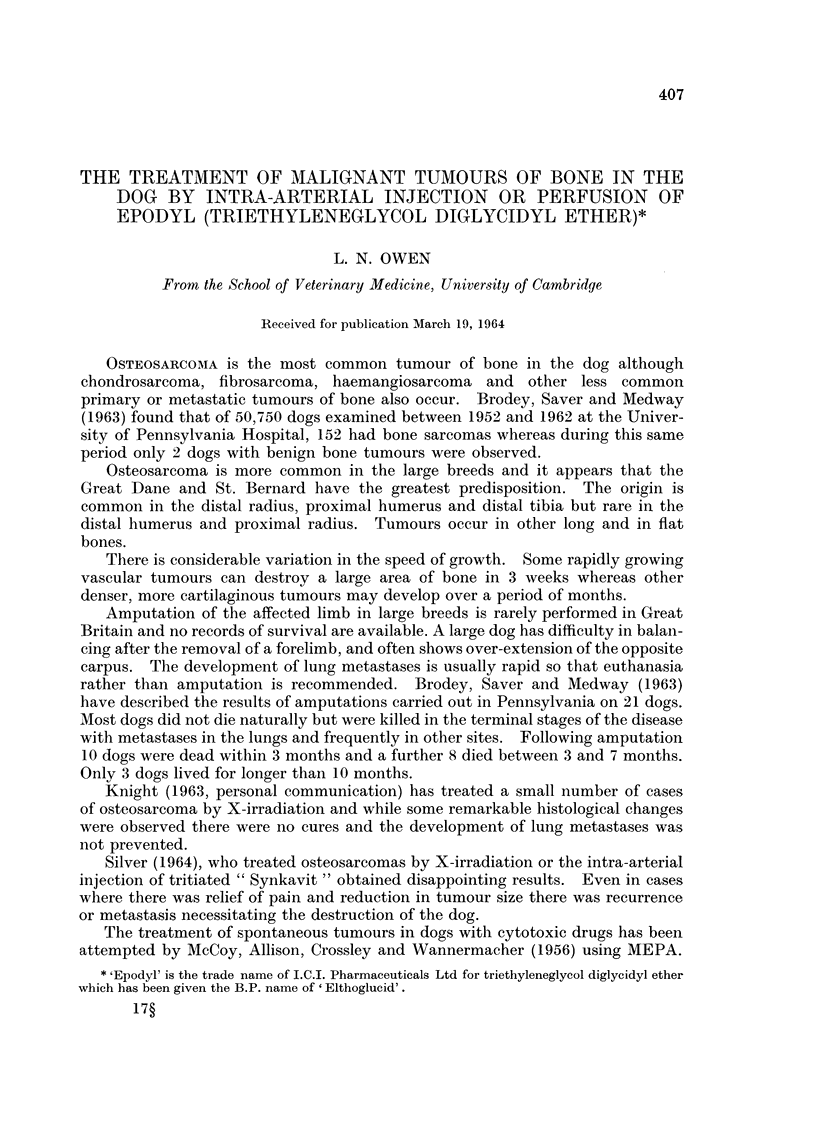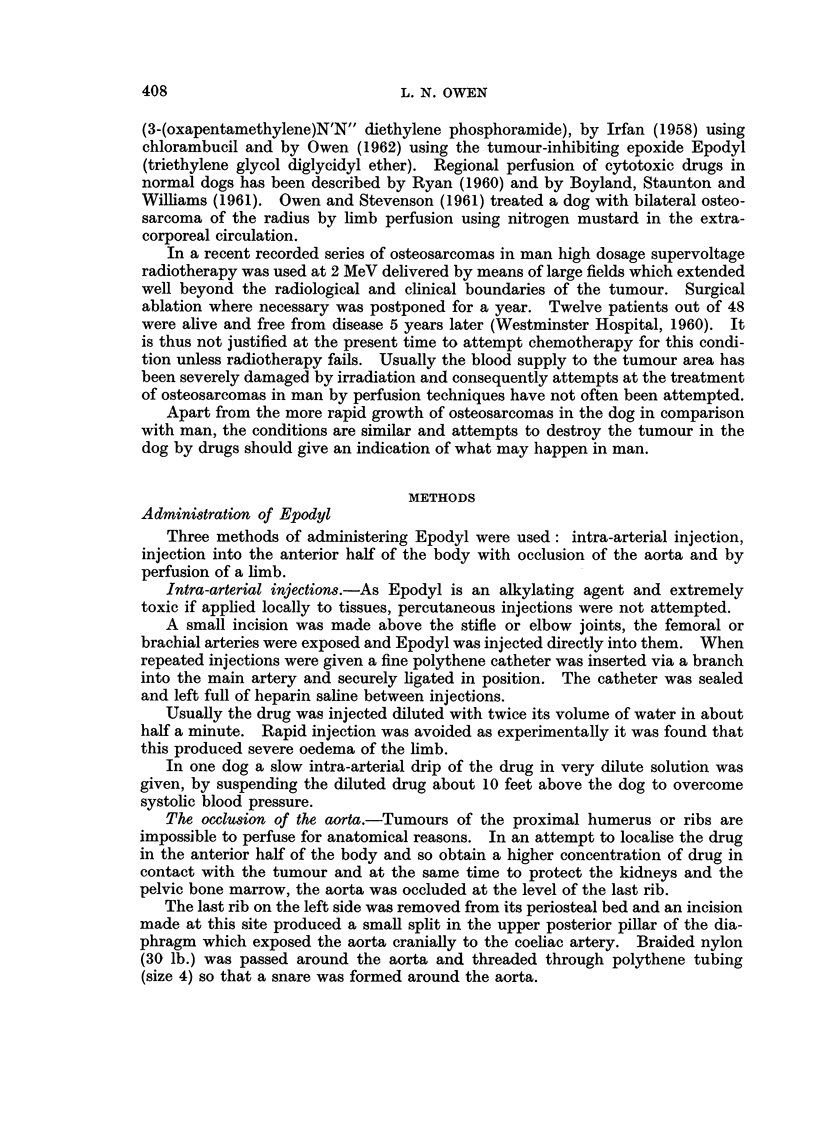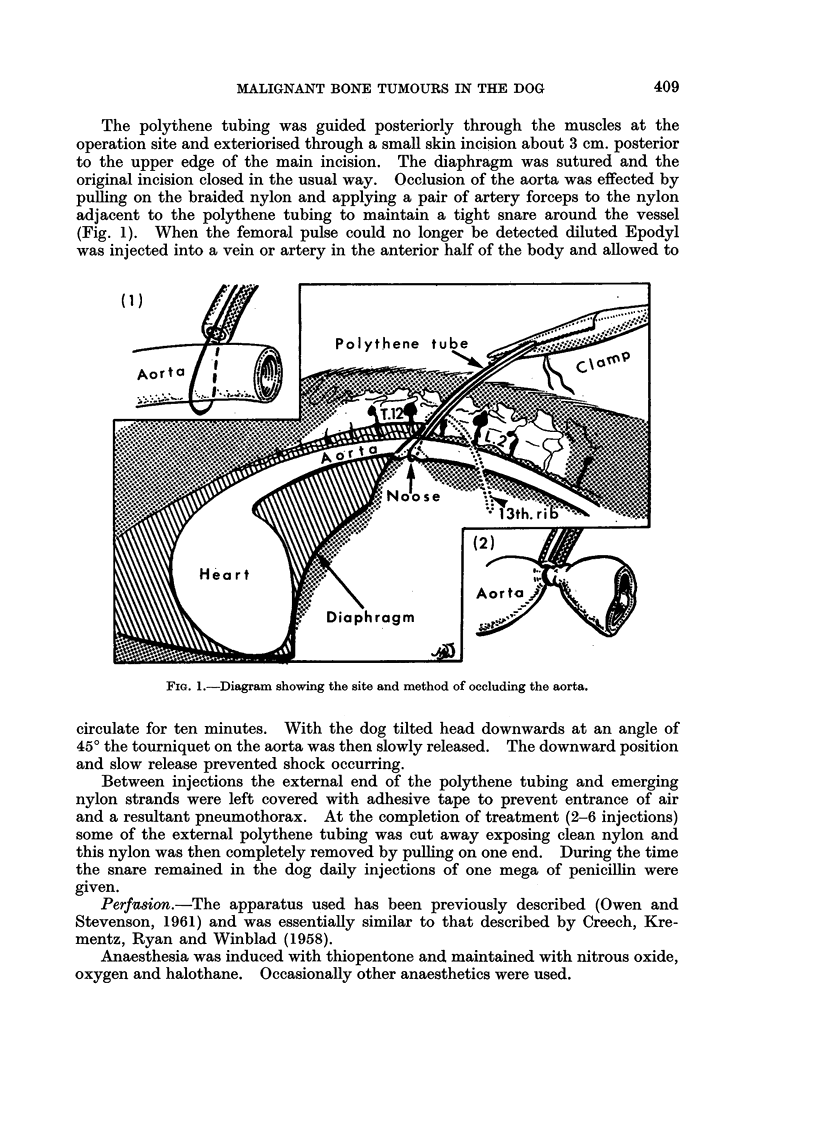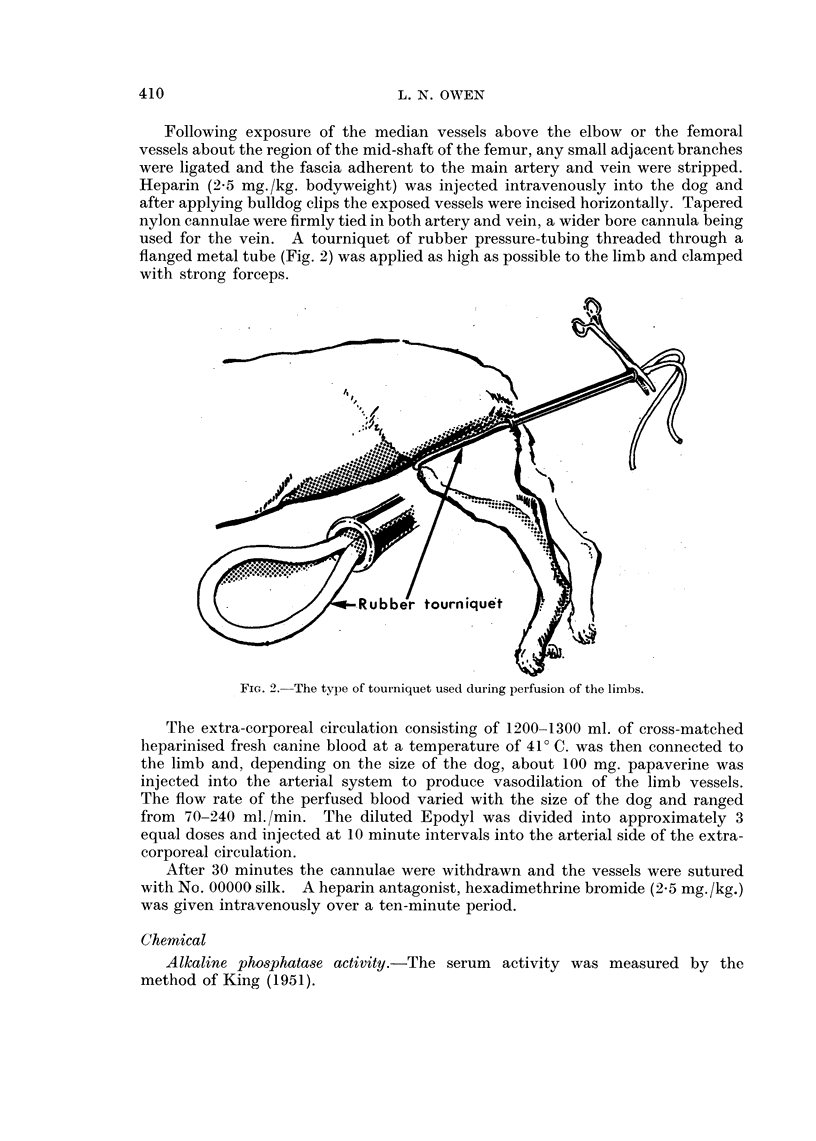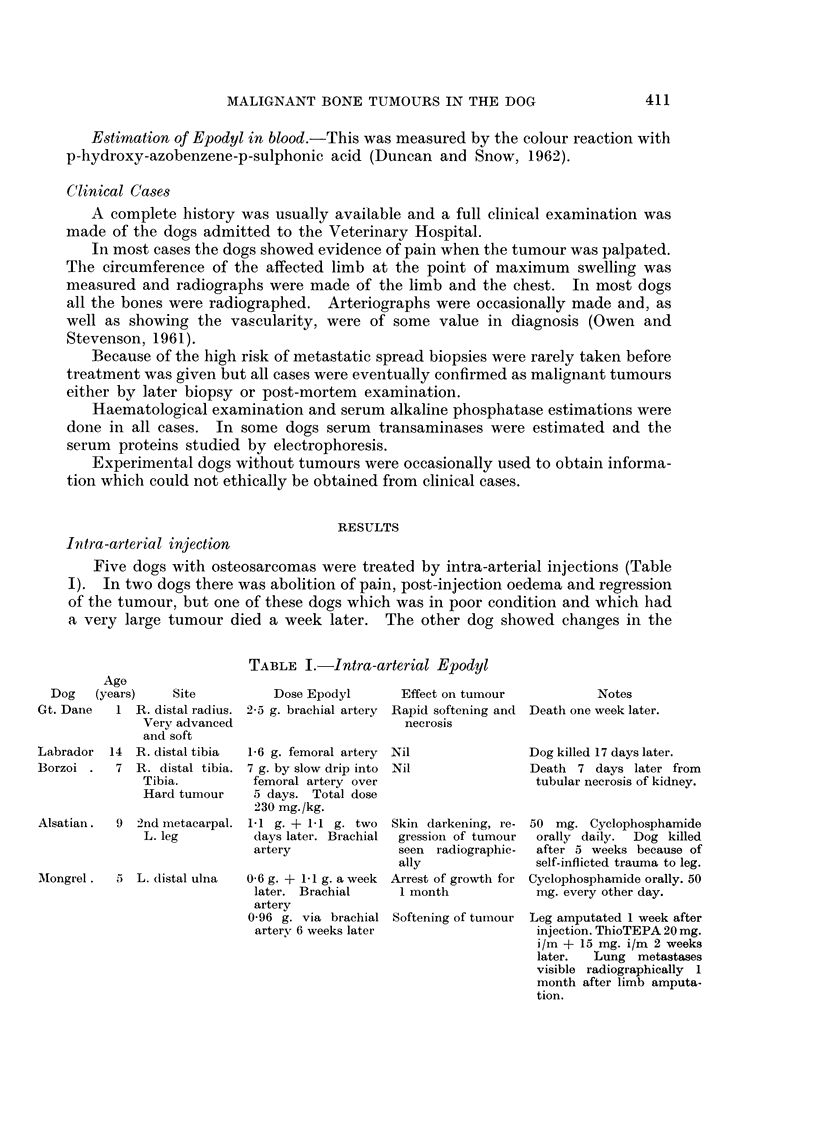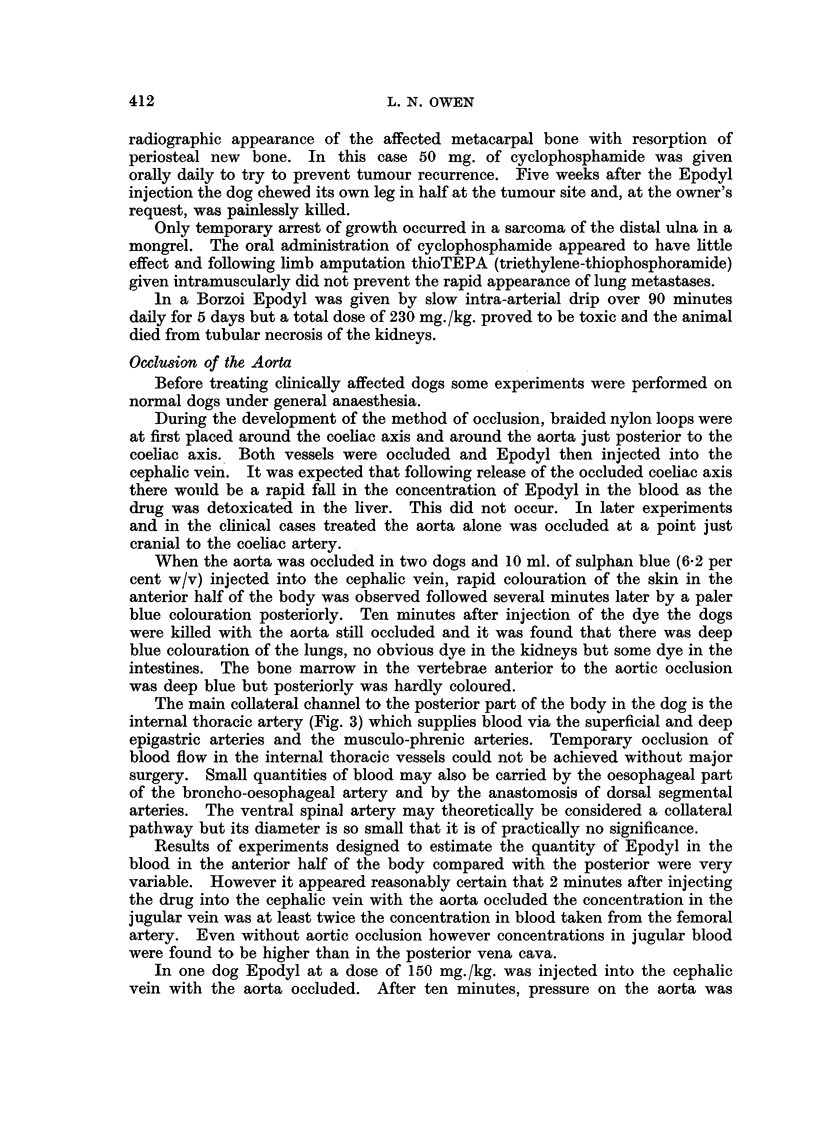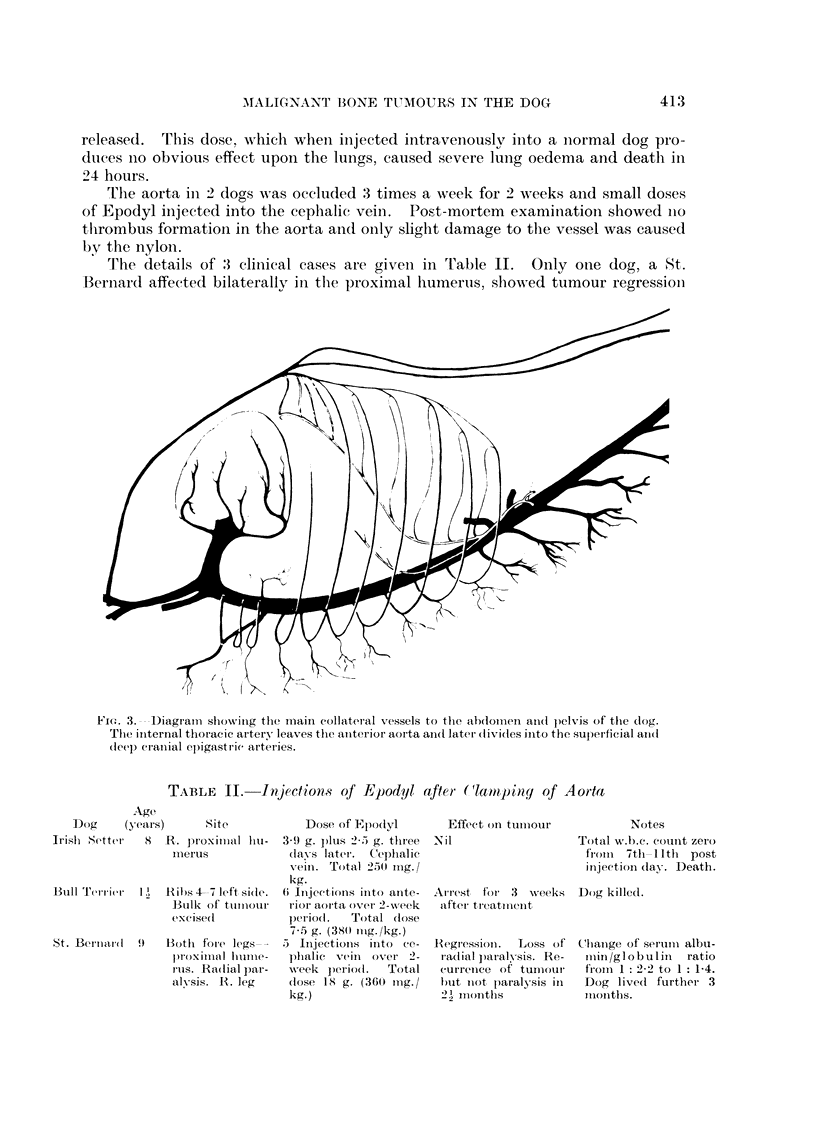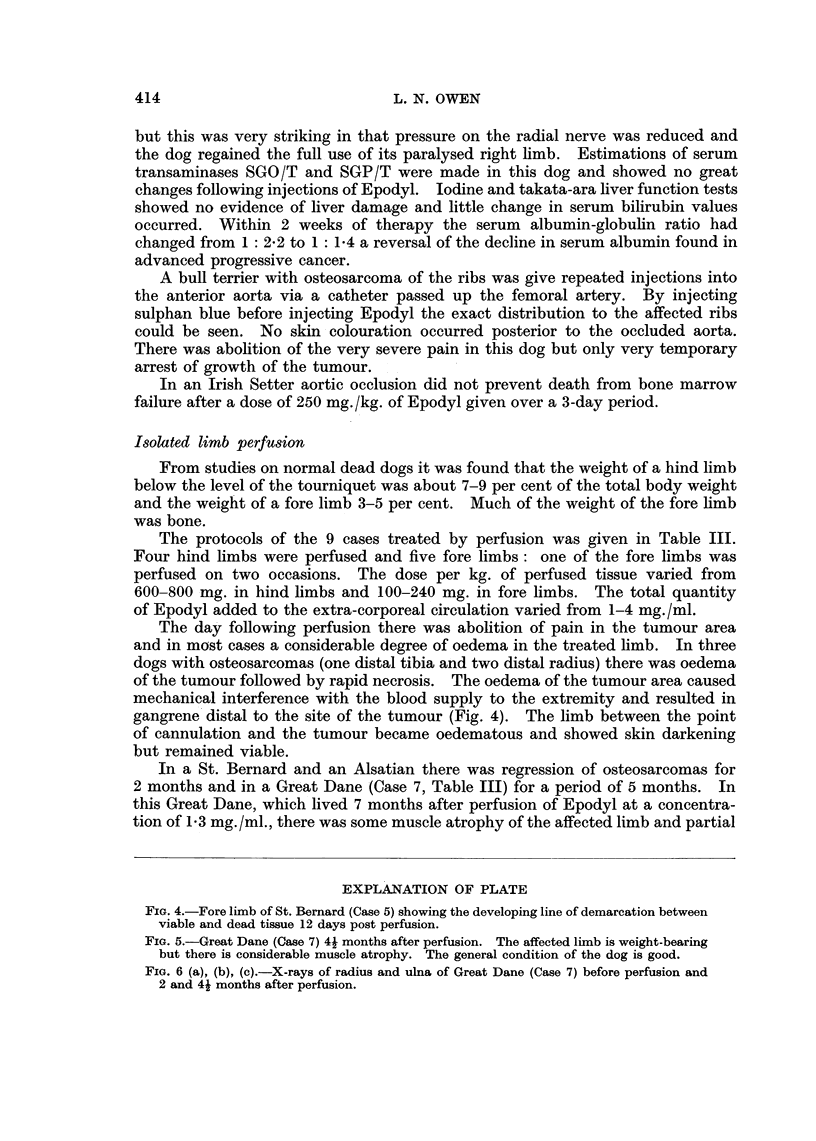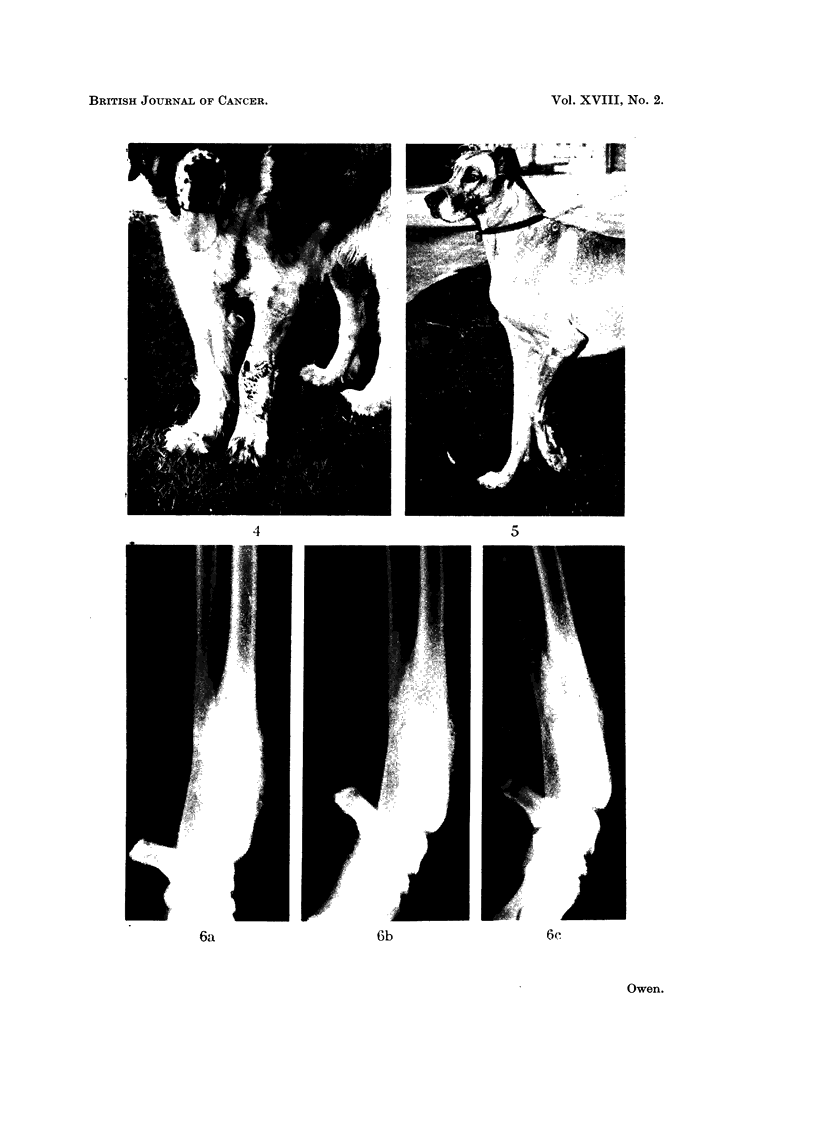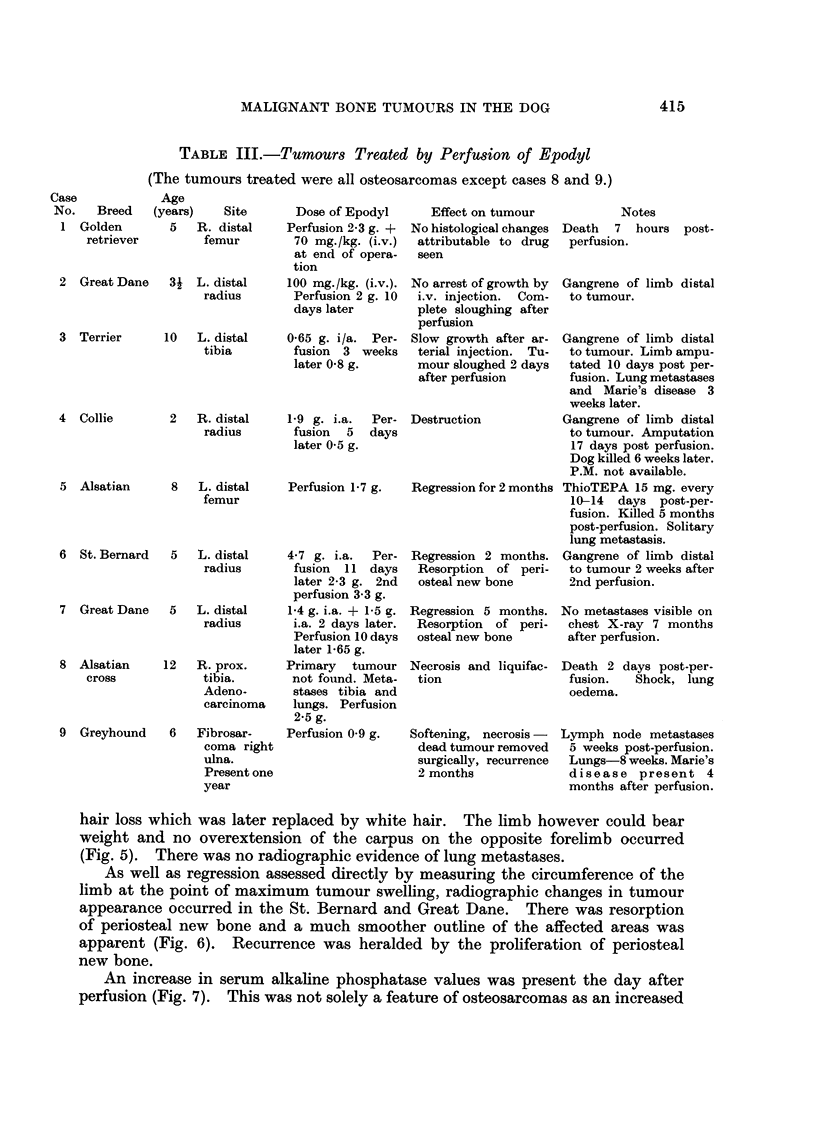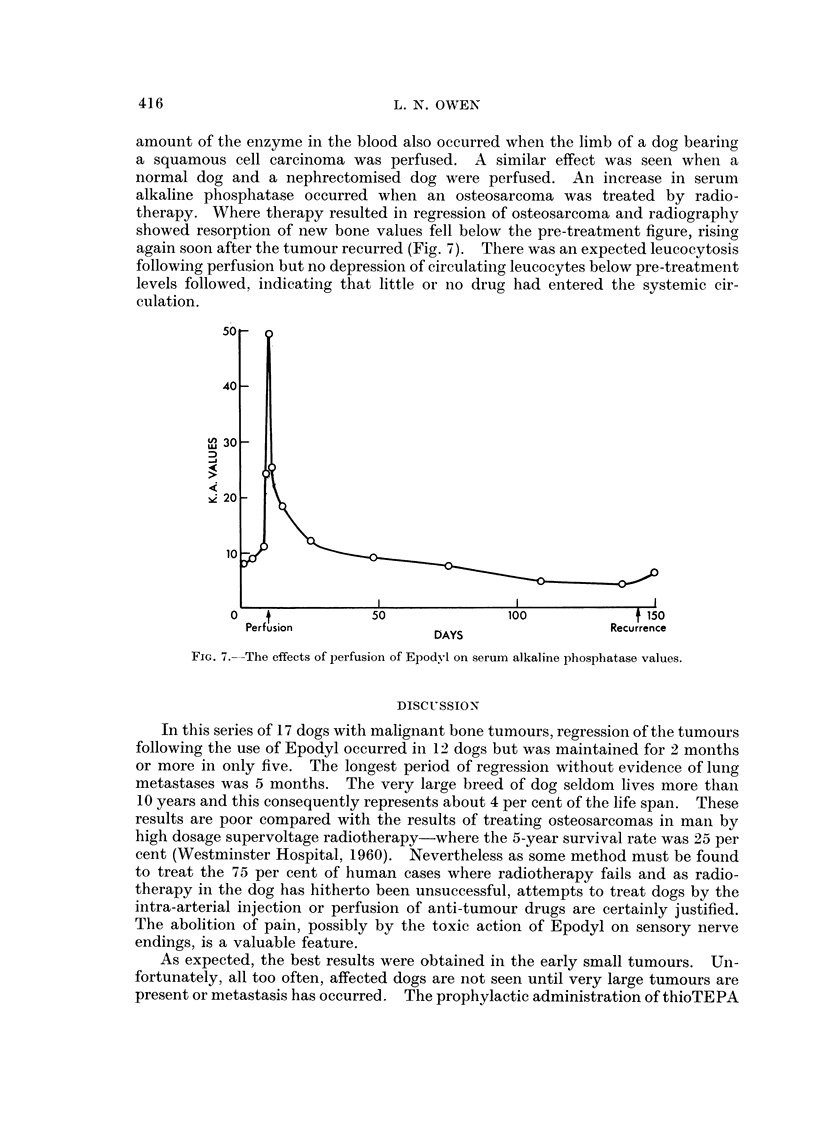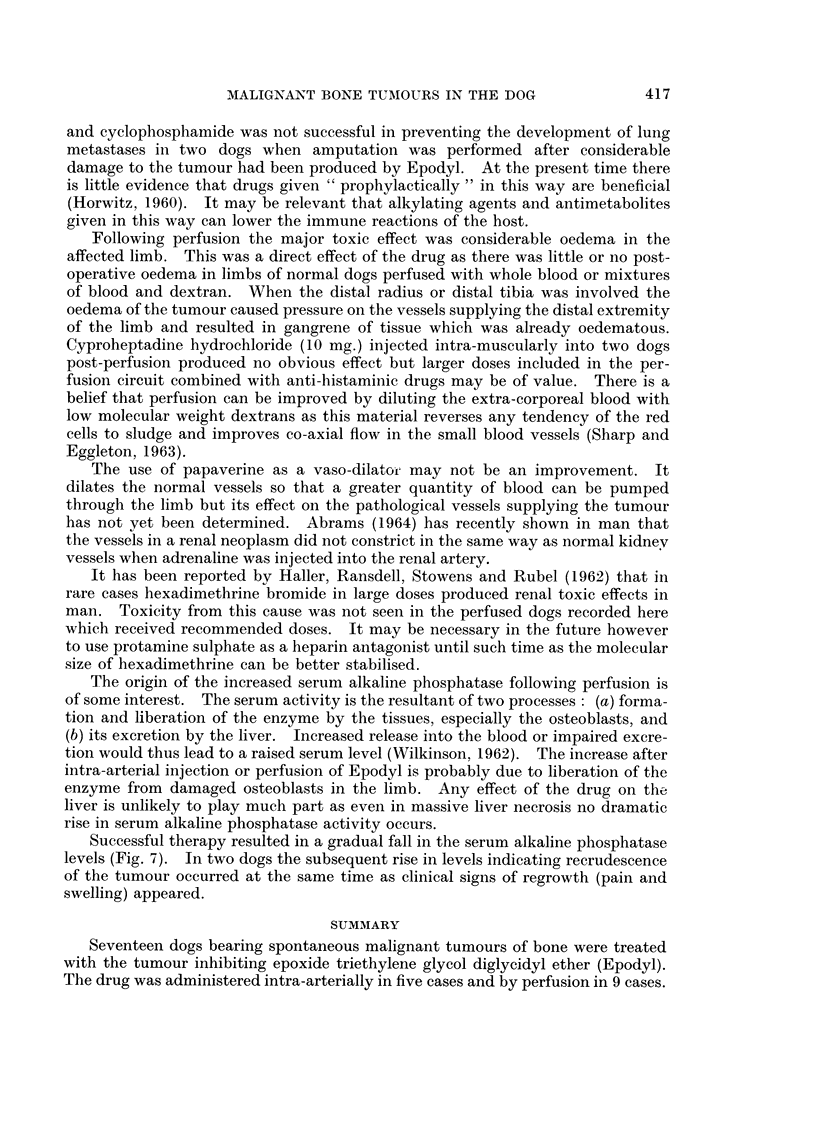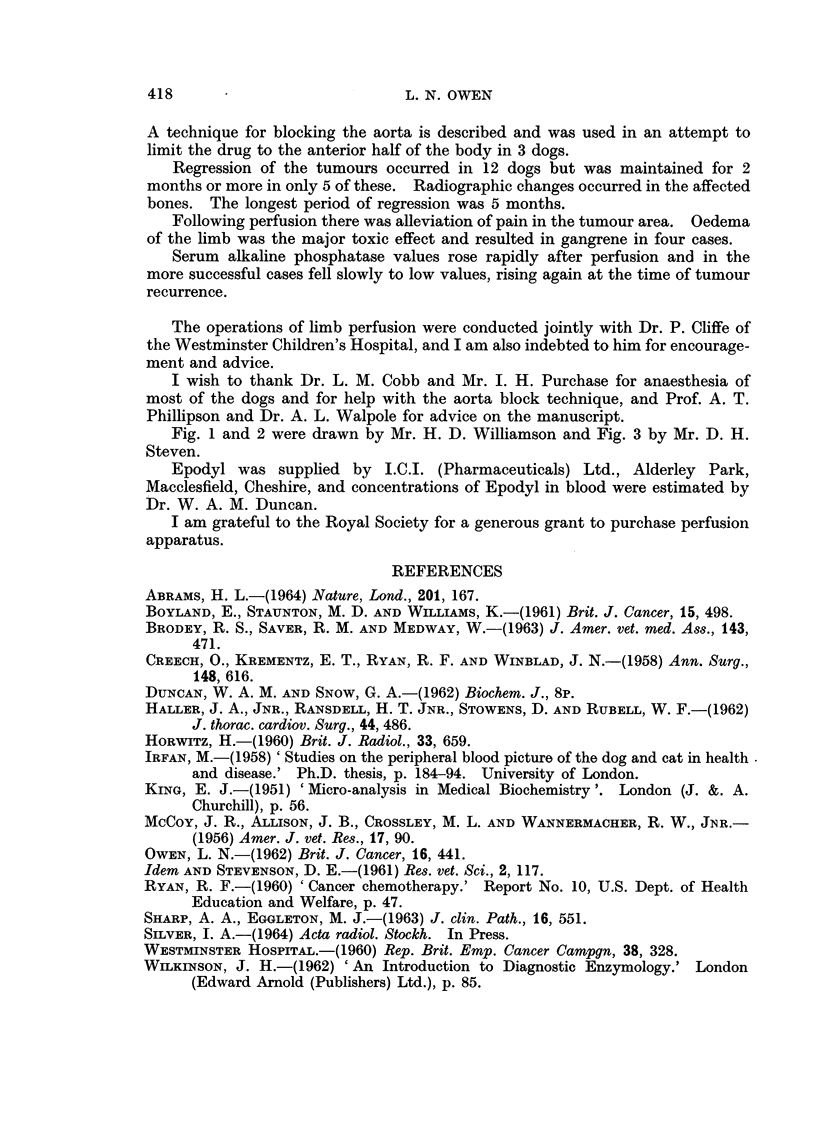# The Treatment of Malignant Tumours of Bone in the Dog by Intra-Arterial Injection or Perfusion of Epodyl (Triethyleneglycol Diglycidyl Ether)*

**DOI:** 10.1038/bjc.1964.47

**Published:** 1964-06

**Authors:** L. N. Owen

## Abstract

**Images:**


### THE TREATMENT OF MALIGNANT TUMOURS OF BONE IN THE DOG BY INTRA-ARTERIAL INJECTION OR PERFUSION OF EPODYL (TRIETHYLENEGLYCOL DIGLYCIDYL ETHER)*

L. N. OWEN

*From the School of Veterinary Medicine, University of Cambridge*

Received for publication March 19, 1964

OSTEOSARCOMA is the most common tumour of bone in the dog although chondrosarcoma, fibrosarcoma, haemangiosarcoma and other less common primary or metastatic tumours of bone also occur. Brodey, Saver and Medway (1963) found that of 50,750 dogs examined between 1952 and 1962 at the University of Pennsylvania Hospital, 152 had bone sarcomas whereas during this same period only 2 dogs with benign bone tumours were observed.

Osteosarcoma is more common in the large breeds and it appears that the Great Dane and St. Bernard have the greatest predisposition. The origin is common in the distal radius, proximal humerus and distal tibia but rare in the distal humerus and proximal radius. Tumours occur in other long and in flat bones.

There is considerable variation in the speed of growth. Some rapidly growing vascular tumours can destroy a large area of bone in 3 weeks whereas other denser, more cartilaginous tumours may develop over a period of months.

Amputation of the affected limb in large breeds is rarely performed in Great Britain and no records of survival are available. A large dog has difficulty in balancing after the removal of a forelimb, and often shows over-extension of the opposite carpus. The development of lung metastases is usually rapid so that euthanasia rather than amputation is recommended. Brodey, Saver and Medway (1963) have described the results of amputations carried out in Pennsylvania on 21 dogs. Most dogs did not die naturally but were killed in the terminal stages of the disease with metastases in the lungs and frequently in other sites. Following amputation 10 dogs were dead within 3 months and a further 8 died between 3 and 7 months. Only 3 dogs lived for longer than 10 months.

Knight (1963, personal communication) has treated a small number of cases of osteosarcoma by X-irradiation and while some remarkable histological changes were observed there were no cures and the development of lung metastases was not prevented.

Silver (1964), who treated osteosarcomas by X-irradiation or the intra-arterial injection of tritiated " Synkavit " obtained disappointing results. Even in cases where there was relief of pain and reduction in tumour size there was recurrence or metastasis necessitating the destruction of the dog.

The treatment of spontaneous tumours in dogs with cytotoxic drugs has been attempted by McCoy, Allison, Crossley and Wannermacher (1956) using MEPA.

* 'Epodyl' is the trade name of I.C.I. Pharmaceuticals Ltd for triethyleneglycol diglycidyl ether which has been given the B.P. name of 'Elthoglucid'.

(3-(oxapentamethylene)N′N″ diethylene phosphoramide), by Irfan (1958) using chlorambucil and by Owen (1962) using the tumour-inhibiting epoxide Epodyl (triethylene glycol diglycidyl ether). Regional perfusion of cytotoxic drugs in normal dogs has been described by Ryan (1960) and by Boyland, Staunton and Williams (1961). Owen and Stevenson (1961) treated a dog with bilateral osteosarcoma of the radius by limb perfusion using nitrogen mustard in the extracorporeal circulation.

In a recent recorded series of osteosarcomas in man high dosage supervoltage radiotherapy was used at 2 MeV delivered by means of large fields which extended well beyond the radiological and clinical boundaries of the tumour. Surgical ablation where necessary was postponed for a year. Twelve patients out of 48 were alive and free from disease 5 years later (Westminster Hospital, 1960). It is thus not justified at the present time to attempt chemotherapy for this condition unless radiotherapy fails. Usually the blood supply to the tumour area has been severely damaged by irradiation and consequently attempts at the treatment of osteosarcomas in man by perfusion techniques have not often been attempted.

Apart from the more rapid growth of osteosarcomas in the dog in comparison with man, the conditions are similar and attempts to destroy the tumour in the dog by drugs should give an indication of what may happen in man.

### METHODS

### *Administration of Epodyl*

Three methods of administering Epodyl were used : intra-arterial injection, injection into the anterior half of the body with occlusion of the aorta and by perfusion of a limb.

*Intra-arterial injections.*—As Epodyl is an alkylating agent and extremely toxic if applied locally to tissues, percutaneous injections were not attempted.

A small incision was made above the stifle or elbow joints, the femoral or brachial arteries were exposed and Epodyl was injected directly into them. When repeated injections were given a fine polythene catheter was inserted via a branch into the main artery and securely ligated in position. The catheter was sealed and left full of heparin saline between injections.

Usually the drug was injected diluted with twice its volume of water in about half a minute. Rapid injection was avoided as experimentally it was found that this produced severe oedema of the limb.

In one dog a slow intra-arterial drip of the drug in very dilute solution was given, by suspending the diluted drug about 10 feet above the dog to overcome systolic blood pressure.

*The occlusion of the aorta.*—Tumours of the proximal humerus or ribs are impossible to perfuse for anatomical reasons. In an attempt to localise the drug in the anterior half of the body and so obtain a higher concentration of drug in contact with the tumour and at the same time to protect the kidneys and the pelvic bone marrow, the aorta was occluded at the level of the last rib.

The last rib on the left side was removed from its periosteal bed and an incision made at this site produced a small split in the upper posterior pillar of the diaphragm which exposed the aorta cranially to the coeliac artery. Braided nylon (30 lb.) was passed around the aorta and threaded through polythene tubing (size 4) so that a snare was formed around the aorta.

The polythene tubing was guided posteriorly through the muscles at the operation site and exteriorised through a small skin incision about 3 cm. posterior to the upper edge of the main incision. The diaphragm was sutured and the original incision closed in the usual way. Occlusion of the aorta was effected by pulling on the braided nylon and applying a pair of artery forceps to the nylon adjacent to the polythene tubing to maintain a tight snare around the vessel (Fig. 1). When the femoral pulse could no longer be detected diluted Epodyl was injected into a vein or artery in the anterior half of the body and allowed to

FIG. 1.—Diagram showing the site and method of occluding the aorta.

circulate for ten minutes. With the dog tilted head downwards at an angle of 45° the tourniquet on the aorta was then slowly released. The downward position and slow release prevented shock occurring.

Between injections the external end of the polythene tubing and emerging nylon strands were left covered with adhesive tape to prevent entrance of air and a resultant pneumothorax. At the completion of treatment (2–6 injections) some of the external polythene tubing was cut away exposing clean nylon and this nylon was then completely removed by pulling on one end. During the time the snare remained in the dog daily injections of one mega of penicillin were given.

*Perfusion.*—The apparatus used has been previously described (Owen and Stevenson, 1961) and was essentially similar to that described by Creech, Krementz, Ryan and Winblad (1958).

Anaesthesia was induced with thiopentone and maintained with nitrous oxide, oxygen and halothane. Occasionally other anaesthetics were used.

Following exposure of the median vessels above the elbow or the femoral vessels about the region of the mid-shaft of the femur, any small adjacent branches were ligated and the fascia adherent to the main artery and vein were stripped. Heparin (2·5 mg./kg. bodyweight) was injected intravenously into the dog and after applying bulldog clips the exposed vessels were incised horizontally. Tapered nylon cannulae were firmly tied in both artery and vein, a wider bore cannula being used for the vein. A tourniquet of rubber pressure-tubing threaded through a flanged metal tube (Fig. 2) was applied as high as possible to the limb and clamped with strong forceps.

FIG. 2.—The type of tourniquet used during perfusion of the limbs.

The extra-corporeal circulation consisting of 1200–1300 ml. of cross-matched heparinised fresh canine blood at a temperature of 41° C. was then connected to the limb and, depending on the size of the dog, about 100 mg. papaverine was injected into the arterial system to produce vasodilation of the limb vessels. The flow rate of the perfused blood varied with the size of the dog and ranged from 70–240 ml./min. The diluted Epodyl was divided into approximately 3 equal doses and injected at 10 minute intervals into the arterial side of the extra-corporeal circulation.

After 30 minutes the cannulae were withdrawn and the vessels were sutured with No. 00000 silk. A heparin antagonist, hexadimethrine bromide (2·5 mg./kg.) was given intravenously over a ten-minute period.

*Chemical*

*Alkaline phosphatase activity.*—The serum activity was measured by the method of King (1951).

*Estimation of Epodyl in blood.*—This was measured by the colour reaction with p-hydroxy-azobenzene-p-sulphonic acid (Duncan and Snow, 1962).

*Clinical Cases*

A complete history was usually available and a full clinical examination was made of the dogs admitted to the Veterinary Hospital.

In most cases the dogs showed evidence of pain when the tumour was palpated. The circumference of the affected limb at the point of maximum swelling was measured and radiographs were made of the limb and the chest. In most dogs all the bones were radiographed. Arteriographs were occasionally made and, as well as showing the vascularity, were of some value in diagnosis (Owen and Stevenson, 1961).

Because of the high risk of metastatic spread biopsies were rarely taken before treatment was given but all cases were eventually confirmed as malignant tumours either by later biopsy or post-mortem examination.

Haematological examination and serum alkaline phosphatase estimations were done in all cases. In some dogs serum transaminases were estimated and the serum proteins studied by electrophoresis.

Experimental dogs without tumours were occasionally used to obtain information which could not ethically be obtained from clinical cases.

### RESULTS

*Intra-arterial injection*

Five dogs with osteosarcomas were treated by intra-arterial injections (Table I). In two dogs there was abolition of pain, post-injection oedema and regression of the tumour, but one of these dogs which was in poor condition and which had a very large tumour died a week later. The other dog showed changes in the

TABLE I.—*Intra-arterial Epodyl*

| Dog | Age (years) | Site | Dose Epodyl | Effect on tumour | Notes |
|---|---|---|---|---|---|
| Gt. Dane | 1 | R. distal radius. Very advanced and soft | 2·5 g. brachial artery | Rapid softening and necrosis | Death one week later. |
| Labrador | 14 | R. distal tibia | 1·6 g. femoral artery | Nil | Dog killed 17 days later. |
| Borzoi . | 7 | R. distal tibia. Tibia. Hard tumour | 7 g. by slow drip into femoral artery over 5 days. Total dose 230 mg./kg. | Nil | Death 7 days later from tubular necrosis of kidney. |
| Alsatian . | 9 | 2nd metacarpal. L. leg | 1·1 g. + 1·1 g. two days later. Brachial artery | Skin darkening, regression of tumour seen radiographically | 50 mg. Cyclophosphamide orally daily. Dog killed after 5 weeks because of self-inflicted trauma to leg. |
| Mongrel . | 5 | L. distal ulna | 0·6 g. + 1·1 g. a week later. Brachial artery | Arrest of growth for 1 month | Cyclophosphamide orally. 50 mg. every other day. |
| | | | 0·96 g. via brachial artery 6 weeks later | Softening of tumour | Leg amputated 1 week after injection. ThioTEPA 20 mg. i/m + 15 mg. i/m 2 weeks later. Lung metastases visible radiographically 1 month after limb amputation. |

radiographic appearance of the affected metacarpal bone with resorption of periosteal new bone. In this case 50 mg. of cyclophosphamide was given orally daily to try to prevent tumour recurrence. Five weeks after the Epodyl injection the dog chewed its own leg in half at the tumour site and, at the owner's request, was painlessly killed.

Only temporary arrest of growth occurred in a sarcoma of the distal ulna in a mongrel. The oral administration of cyclophosphamide appeared to have little effect and following limb amputation thioTEPA (triethylene-thiophosphoramide) given intramuscularly did not prevent the rapid appearance of lung metastases.

In a Borzoi Epodyl was given by slow intra-arterial drip over 90 minutes daily for 5 days but a total dose of 230 mg./kg. proved to be toxic and the animal died from tubular necrosis of the kidneys.

*Occlusion of the Aorta*

Before treating clinically affected dogs some experiments were performed on normal dogs under general anaesthesia.

During the development of the method of occlusion, braided nylon loops were at first placed around the coeliac axis and around the aorta just posterior to the coeliac axis. Both vessels were occluded and Epodyl then injected into the cephalic vein. It was expected that following release of the occluded coeliac axis there would be a rapid fall in the concentration of Epodyl in the blood as the drug was detoxicated in the liver. This did not occur. In later experiments and in the clinical cases treated the aorta alone was occluded at a point just cranial to the coeliac artery.

When the aorta was occluded in two dogs and 10 ml. of sulphan blue (6·2 per cent w/v) injected into the cephalic vein, rapid colouration of the skin in the anterior half of the body was observed followed several minutes later by a paler blue colouration posteriorly. Ten minutes after injection of the dye the dogs were killed with the aorta still occluded and it was found that there was deep blue colouration of the lungs, no obvious dye in the kidneys but some dye in the intestines. The bone marrow in the vertebrae anterior to the aortic occlusion was deep blue but posteriorly was hardly coloured.

The main collateral channel to the posterior part of the body in the dog is the internal thoracic artery (Fig. 3) which supplies blood via the superficial and deep epigastric arteries and the musculo-phrenic arteries. Temporary occlusion of blood flow in the internal thoracic vessels could not be achieved without major surgery. Small quantities of blood may also be carried by the oesophageal part of the broncho-oesophageal artery and by the anastomosis of dorsal segmental arteries. The ventral spinal artery may theoretically be considered a collateral pathway but its diameter is so small that it is of practically no significance.

Results of experiments designed to estimate the quantity of Epodyl in the blood in the anterior half of the body compared with the posterior were very variable. However it appeared reasonably certain that 2 minutes after injecting the drug into the cephalic vein with the aorta occluded the concentration in the jugular vein was at least twice the concentration in blood taken from the femoral artery. Even without aortic occlusion however concentrations in jugular blood were found to be higher than in the posterior vena cava.

In one dog Epodyl at a dose of 150 mg./kg. was injected into the cephalic vein with the aorta occluded. After ten minutes, pressure on the aorta was released. This dose, which when injected intravenously into a normal dog produces no obvious effect upon the lungs, caused severe lung oedema and death in 24 hours.

The aorta in 2 dogs was occluded 3 times a week for 2 weeks and small doses of Epodyl injected into the cephalic vein. Post-mortem examination showed no thrombus formation in the aorta and only slight damage to the vessel was caused by the nylon.

The details of 3 clinical cases are given in Table II. Only one dog, a St. Bernard affected bilaterally in the proximal humerus, showed tumour regression

FIG. 3.—Diagram showing the main collateral vessels to the abdomen and pelvis of the dog. The internal thoracic artery leaves the anterior aorta and later divides into the superficial and deep cranial epigastric arteries.

TABLE II.—*Injections of Epodyl after Clamping of Aorta*

| Dog | Age (years) | Site | Dose of Epodyl | Effect on tumour | Notes |
|---|---|---|---|---|---|
| Irish Setter | 8 | R. proximal humerus | 3·9 g. plus 2·5 g. three days later. Cephalic vein. Total 250 mg./kg. | Nil | Total w.b.c. count zero from 7th–11th post injection day. Death. |
| Bull Terrier | $1\frac{1}{2}$ | Ribs 4–7 left side. Bulk of tumour excised | 6 Injections into anterior aorta over 2-week period. Total dose 7·5 g. (380 mg./kg.) | Arrest for 3 weeks after treatment | Dog killed. |
| St. Bernard | 9 | Both fore legs—proximal humerus. Radial paralysis. R. leg | 5 Injections into cephalic vein over 2-week period. Total dose 18 g. (360 mg./kg.) | Regression. Loss of radial paralysis. Recurrence of tumour but not paralysis in $2\frac{1}{2}$ months | Change of serum albumin/globulin ratio from 1 : 2·2 to 1 : 1·4. Dog lived further 3 months. |

but this was very striking in that pressure on the radial nerve was reduced and the dog regained the full use of its paralysed right limb. Estimations of serum transaminases SGO/T and SGP/T were made in this dog and showed no great changes following injections of Epodyl. Iodine and takata-ara liver function tests showed no evidence of liver damage and little change in serum bilirubin values occurred. Within 2 weeks of therapy the serum albumin-globulin ratio had changed from 1 : 2·2 to 1 : 1·4 a reversal of the decline in serum albumin found in advanced progressive cancer.

A bull terrier with osteosarcoma of the ribs was give repeated injections into the anterior aorta via a catheter passed up the femoral artery. By injecting sulphan blue before injecting Epodyl the exact distribution to the affected ribs could be seen. No skin colouration occurred posterior to the occluded aorta. There was abolition of the very severe pain in this dog but only very temporary arrest of growth of the tumour.

In an Irish Setter aortic occlusion did not prevent death from bone marrow failure after a dose of 250 mg./kg. of Epodyl given over a 3-day period.

*Isolated limb perfusion*

From studies on normal dead dogs it was found that the weight of a hind limb below the level of the tourniquet was about 7–9 per cent of the total body weight and the weight of a fore limb 3–5 per cent. Much of the weight of the fore limb was bone.

The protocols of the 9 cases treated by perfusion was given in Table III. Four hind limbs were perfused and five fore limbs : one of the fore limbs was perfused on two occasions. The dose per kg. of perfused tissue varied from 600–800 mg. in hind limbs and 100–240 mg. in fore limbs. The total quantity of Epodyl added to the extra-corporeal circulation varied from 1–4 mg./ml.

The day following perfusion there was abolition of pain in the tumour area and in most cases a considerable degree of oedema in the treated limb. In three dogs with osteosarcomas (one distal tibia and two distal radius) there was oedema of the tumour followed by rapid necrosis. The oedema of the tumour area caused mechanical interference with the blood supply to the extremity and resulted in gangrene distal to the site of the tumour (Fig. 4). The limb between the point of cannulation and the tumour became oedematous and showed skin darkening but remained viable.

In a St. Bernard and an Alsatian there was regression of osteosarcomas for 2 months and in a Great Dane (Case 7, Table III) for a period of 5 months. In this Great Dane, which lived 7 months after perfusion of Epodyl at a concentration of 1·3 mg./ml., there was some muscle atrophy of the affected limb and partial

---

EXPLANATION OF PLATE

FIG. 4.—Fore limb of St. Bernard (Case 5) showing the developing line of demarcation between viable and dead tissue 12 days post perfusion.

FIG. 5.—Great Dane (Case 7) 4½ months after perfusion. The affected limb is weight-bearing but there is considerable muscle atrophy. The general condition of the dog is good.

FIG. 6 (a), (b), (c).—X-rays of radius and ulna of Great Dane (Case 7) before perfusion and 2 and 4½ months after perfusion.

4

5

6a

6b

6c

TABLE III.—*Tumours Treated by Perfusion of Epodyl*

(The tumours treated were all osteosarcomas except cases 8 and 9.)

| Case No. | Breed | Age (years) | Site | Dose of Epodyl | Effect on tumour | Notes |
|---|---|---|---|---|---|---|
| 1 | Golden retriever | 5 | R. distal femur | Perfusion 2·3 g. + 70 mg./kg. (i.v.) at end of operation | No histological changes attributable to drug seen | Death 7 hours post-perfusion. |
| 2 | Great Dane | 3½ | L. distal radius | 100 mg./kg. (i.v.). Perfusion 2 g. 10 days later | No arrest of growth by i.v. injection. Complete sloughing after perfusion | Gangrene of limb distal to tumour. |
| 3 | Terrier | 10 | L. distal tibia | 0·65 g. i/a. Perfusion 3 weeks later 0·8 g. | Slow growth after arterial injection. Tumour sloughed 2 days after perfusion | Gangrene of limb distal to tumour. Limb amputated 10 days post perfusion. Lung metastases and Marie's disease 3 weeks later. |
| 4 | Collie | 2 | R. distal radius | 1·9 g. i.a. Perfusion 5 days later 0·5 g. | Destruction | Gangrene of limb distal to tumour. Amputation 17 days post perfusion. Dog killed 6 weeks later. P.M. not available. |
| 5 | Alsatian | 8 | L. distal femur | Perfusion 1·7 g. | Regression for 2 months | ThioTEPA 15 mg. every 10–14 days post-perfusion. Killed 5 months post-perfusion. Solitary lung metastasis. |
| 6 | St. Bernard | 5 | L. distal radius | 4·7 g. i.a. Perfusion 11 days later 2·3 g. 2nd perfusion 3·3 g. | Regression 2 months. Resorption of periosteal new bone | Gangrene of limb distal to tumour 2 weeks after 2nd perfusion. |
| 7 | Great Dane | 5 | L. distal radius | 1·4 g. i.a. + 1·5 g. i.a. 2 days later. Perfusion 10 days later 1·65 g. | Regression 5 months. Resorption of periosteal new bone | No metastases visible on chest X-ray 7 months after perfusion. |
| 8 | Alsatian cross | 12 | R. prox. tibia. Adenocarcinoma | Primary tumour not found. Metastases tibia and lungs. Perfusion 2·5 g. | Necrosis and liquifaction | Death 2 days post-perfusion. Shock, lung oedema. |
| 9 | Greyhound | 6 | Fibrosarcoma right ulna. Present one year | Perfusion 0·9 g. | Softening, necrosis — dead tumour removed surgically, recurrence 2 months | Lymph node metastases 5 weeks post-perfusion. Lungs—8 weeks. Marie's disease present 4 months after perfusion. |

hair loss which was later replaced by white hair. The limb however could bear weight and no overextension of the carpus on the opposite forelimb occurred (Fig. 5). There was no radiographic evidence of lung metastases.

As well as regression assessed directly by measuring the circumference of the limb at the point of maximum tumour swelling, radiographic changes in tumour appearance occurred in the St. Bernard and Great Dane. There was resorption of periosteal new bone and a much smoother outline of the affected areas was apparent (Fig. 6). Recurrence was heralded by the proliferation of periosteal new bone.

An increase in serum alkaline phosphatase values was present the day after perfusion (Fig. 7). This was not solely a feature of osteosarcomas as an increased amount of the enzyme in the blood also occurred when the limb of a dog bearing a squamous cell carcinoma was perfused. A similar effect was seen when a normal dog and a nephrectomised dog were perfused. An increase in serum alkaline phosphatase occurred when an osteosarcoma was treated by radiotherapy. Where therapy resulted in regression of osteosarcoma and radiography showed resorption of new bone values fell below the pre-treatment figure, rising again soon after the tumour recurred (Fig. 7). There was an expected leucocytosis following perfusion but no depression of circulating leucocytes below pre-treatment levels followed, indicating that little or no drug had entered the systemic circulation.

Fig. 7.—The effects of perfusion of Epodyl on serum alkaline phosphatase values.

### DISCUSSION

In this series of 17 dogs with malignant bone tumours, regression of the tumours following the use of Epodyl occurred in 12 dogs but was maintained for 2 months or more in only five. The longest period of regression without evidence of lung metastases was 5 months. The very large breed of dog seldom lives more than 10 years and this consequently represents about 4 per cent of the life span. These results are poor compared with the results of treating osteosarcomas in man by high dosage supervoltage radiotherapy—where the 5-year survival rate was 25 per cent (Westminster Hospital, 1960). Nevertheless as some method must be found to treat the 75 per cent of human cases where radiotherapy fails and as radiotherapy in the dog has hitherto been unsuccessful, attempts to treat dogs by the intra-arterial injection or perfusion of anti-tumour drugs are certainly justified. The abolition of pain, possibly by the toxic action of Epodyl on sensory nerve endings, is a valuable feature.

As expected, the best results were obtained in the early small tumours. Unfortunately, all too often, affected dogs are not seen until very large tumours are present or metastasis has occurred. The prophylactic administration of thioTEPA

and cyclophosphamide was not successful in preventing the development of lung metastases in two dogs when amputation was performed after considerable damage to the tumour had been produced by Epodyl. At the present time there is little evidence that drugs given " prophylactically " in this way are beneficial (Horwitz, 1960). It may be relevant that alkylating agents and antimetabolites given in this way can lower the immune reactions of the host.

Following perfusion the major toxic effect was considerable oedema in the affected limb. This was a direct effect of the drug as there was little or no post-operative oedema in limbs of normal dogs perfused with whole blood or mixtures of blood and dextran. When the distal radius or distal tibia was involved the oedema of the tumour caused pressure on the vessels supplying the distal extremity of the limb and resulted in gangrene of tissue which was already oedematous. Cyproheptadine hydrochloride (10 mg.) injected intra-muscularly into two dogs post-perfusion produced no obvious effect but larger doses included in the perfusion circuit combined with anti-histaminic drugs may be of value. There is a belief that perfusion can be improved by diluting the extra-corporeal blood with low molecular weight dextrans as this material reverses any tendency of the red cells to sludge and improves co-axial flow in the small blood vessels (Sharp and Eggleton, 1963).

The use of papaverine as a vaso-dilator may not be an improvement. It dilates the normal vessels so that a greater quantity of blood can be pumped through the limb but its effect on the pathological vessels supplying the tumour has not yet been determined. Abrams (1964) has recently shown in man that the vessels in a renal neoplasm did not constrict in the same way as normal kidney vessels when adrenaline was injected into the renal artery.

It has been reported by Haller, Ransdell, Stowens and Rubel (1962) that in rare cases hexadimethrine bromide in large doses produced renal toxic effects in man. Toxicity from this cause was not seen in the perfused dogs recorded here which received recommended doses. It may be necessary in the future however to use protamine sulphate as a heparin antagonist until such time as the molecular size of hexadimethrine can be better stabilised.

The origin of the increased serum alkaline phosphatase following perfusion is of some interest. The serum activity is the resultant of two processes : (*a*) formation and liberation of the enzyme by the tissues, especially the osteoblasts, and (*b*) its excretion by the liver. Increased release into the blood or impaired excretion would thus lead to a raised serum level (Wilkinson, 1962). The increase after intra-arterial injection or perfusion of Epodyl is probably due to liberation of the enzyme from damaged osteoblasts in the limb. Any effect of the drug on the liver is unlikely to play much part as even in massive liver necrosis no dramatic rise in serum alkaline phosphatase activity occurs.

Successful therapy resulted in a gradual fall in the serum alkaline phosphatase levels (Fig. 7). In two dogs the subsequent rise in levels indicating recrudescence of the tumour occurred at the same time as clinical signs of regrowth (pain and swelling) appeared.

### SUMMARY

Seventeen dogs bearing spontaneous malignant tumours of bone were treated with the tumour inhibiting epoxide triethylene glycol diglycidyl ether (Epodyl). The drug was administered intra-arterially in five cases and by perfusion in 9 cases.

A technique for blocking the aorta is described and was used in an attempt to limit the drug to the anterior half of the body in 3 dogs.

Regression of the tumours occurred in 12 dogs but was maintained for 2 months or more in only 5 of these. Radiographic changes occurred in the affected bones. The longest period of regression was 5 months.

Following perfusion there was alleviation of pain in the tumour area. Oedema of the limb was the major toxic effect and resulted in gangrene in four cases.

Serum alkaline phosphatase values rose rapidly after perfusion and in the more successful cases fell slowly to low values, rising again at the time of tumour recurrence.

The operations of limb perfusion were conducted jointly with Dr. P. Cliffe of the Westminster Children's Hospital, and I am also indebted to him for encouragement and advice.

I wish to thank Dr. L. M. Cobb and Mr. I. H. Purchase for anaesthesia of most of the dogs and for help with the aorta block technique, and Prof. A. T. Phillipson and Dr. A. L. Walpole for advice on the manuscript.

Fig. 1 and 2 were drawn by Mr. H. D. Williamson and Fig. 3 by Mr. D. H. Steven.

Epodyl was supplied by I.C.I. (Pharmaceuticals) Ltd., Alderley Park, Macclesfield, Cheshire, and concentrations of Epodyl in blood were estimated by Dr. W. A. M. Duncan.

I am grateful to the Royal Society for a generous grant to purchase perfusion apparatus.

### REFERENCES

Abrams, H. L.—(1964) *Nature, Lond.*, **201**, 167.

Boyland, E., Staunton, M. D. and Williams, K.—(1961) *Brit. J. Cancer*, **15**, 498.

Brodey, R. S., Saver, R. M. and Medway, W.—(1963) *J. Amer. vet. med. Ass.*, **143**, 471.

Creech, O., Krementz, E. T., Ryan, R. F. and Winblad, J. N.—(1958) *Ann. Surg.*, **148**, 616.

Duncan, W. A. M. and Snow, G. A.—(1962) *Biochem. J.*, 8P.

Haller, J. A., Jnr., Ransdell, H. T. Jnr., Stowens, D. and Rubell, W. F.—(1962) *J. thorac. cardiov. Surg.*, **44**, 486.

Horwitz, H.—(1960) *Brit. J. Radiol.*, **33**, 659.

Irfan, M.—(1958) 'Studies on the peripheral blood picture of the dog and cat in health and disease.' Ph.D. thesis, p. 184–94. University of London.

King, E. J.—(1951) 'Micro-analysis in Medical Biochemistry'. London (J. &. A. Churchill), p. 56.

McCoy, J. R., Allison, J. B., Crossley, M. L. and Wannermacher, R. W., Jnr.—(1956) *Amer. J. vet. Res.*, **17**, 90.

Owen, L. N.—(1962) *Brit. J. Cancer*, **16**, 441.

*Idem* and Stevenson, D. E.—(1961) *Res. vet. Sci.*, **2**, 117.

Ryan, R. F.—(1960) 'Cancer chemotherapy.' Report No. 10, U.S. Dept. of Health Education and Welfare, p. 47.

Sharp, A. A., Eggleton, M. J.—(1963) *J. clin. Path.*, **16**, 551.

Silver, I. A.—(1964) *Acta radiol. Stockh.* In Press.

Westminster Hospital.—(1960) *Rep. Brit. Emp. Cancer Campgn*, **38**, 328.

Wilkinson, J. H.—(1962) 'An Introduction to Diagnostic Enzymology.' London (Edward Arnold (Publishers) Ltd.), p. 85.